# Korean Medicine in General Practice: Current Status, Challenges, and Vision in Clinical Evidence

**DOI:** 10.1155/2016/3269474

**Published:** 2016-04-27

**Authors:** Tae-Hun Kim, Christopher Zaslawski, Sunoh Kwon, Jung Won Kang

**Affiliations:** ^1^Korean Medicine Clinical Trial Center, Korean Medicine Hospital, Kyung Hee University, Seoul 02447, Republic of Korea; ^2^University of Technology, Sydney, NSW, Australia; ^3^Department of Psychiatry and Behavioral Sciences, Northwestern University Feinberg School of Medicine, Chicago, IL 60611, USA; ^4^Department of Acupuncture & Moxibustion, College of Korean Medicine, Kyung Hee University, Seoul 02447, Republic of Korea

Research mirrors the reality of clinical practice in a society. Korean medicine (KM) shares many medical theories, principles, and interventions with other Traditional Eastern Asian Medicines (TEAM) which is due to Korea's interaction over the last two millennia with its geographical neighbors. Korean medicine however has developed specific clinical approaches and characteristics which can be observed when studying Korean medicine practitioners. These distinctive features and characteristics of Korean medicine have arisen due to the unique medical environment and resources shaped by the geological, cultural, and economic forces of ancient and modern Korea. We believe that the unique features of KM have evolved not only through the influence of the larger TEAM regional activity but also through the influence of local regional KM practices.

Since modern biomedicine has become the mainstream medicine in Korea, legislation and regulation have restricted KM practice to classical medical theory and its associated diagnostic framework and traditional treatment approaches. This has meant that KM doctors have been prohibited to use newly developed and conventional diagnostic imaging tools such as computed tomography and ultrasonography as part of their practice. Furthermore, the position of KM doctors continues to be threatened by biomedical doctors using acupuncture-related interventions and phytomedical products which had been previously regarded as the exclusive domain of KM doctors. Considering these circumstances, what can KM doctors do to remedy this situation and prepare for a better future? Research is one solution to advance the position of KM. By undertaking rigorous clinical studies, vague diagnostic concepts and poor clinical practices will wane, and an evidence-based KM approach to clinical practice will arise. This is surely a situation which all parties in the healthcare sector support.

In this special issue, you can examine current KM practice through clinical studies about new understanding of pattern identification (PI), cross-sectional surveys, evidence-based assessment of common KM interventions, and observational studies on the effect of KM on a variety of stubborn diseases.

PI, an important concept in the KM diagnostic framework, was examined from several perspectives. Patients with dyspepsia often express nongastrointestinal symptoms such as cold hands and feet which are important symptoms for the diagnosis of “spleen deficiency” in KM pattern identification. The study by K.-H. Bae et al. evaluated the responses from 6,444 patients and demonstrated a close association between dyspepsia and cold hypersensitivity of hands and feet. In another study, W. Jung et al. reported different clinical outcomes of acute stage stroke patients in accord with the result of PI. This suggests that PI can be a potential tool for predicting the prognosis of some specific diseases. The development of validated and reliable diagnostic questionnaires for PI is an active research area in KM. H. Kim et al. reported the results of a validation study for the “Phlegm Pattern Questionnaire.” Identifying appropriate methods for developing such instruments and establishing models for the statistical analysis will be helpful to improve the diagnostic accuracy for this emerging area of PI questionnaires. Another interesting study by M. M. Ko and H. Kim reanalyzed the coincidence rate of PI for stroke patients as determined by KM doctors and current stroke questionnaires suggesting a new analytic model for lowering misclassification probability. While it is acknowledged that studies like these are preliminary they are valuable for obtaining a better understanding of PI in KM.

From a therapeutic perspective, KM doctors display similar but different treatment approaches compared to practitioners from other countries. Pharmacoacupuncture, a technique that involves acupuncture-point injection with a single herb extract or herbal formula derivatives, is routinely administered as an acupuncture-related intervention by KM doctors. J. Park et al. conducted a systematic review of randomized controlled trials and assessed the clinical evidence of pharmacoacupuncture for a variety of conditions. Another interesting study by K.-J. Yun et al. analyzed the characteristics of the patients who attended a tertiary KM hospital and concluded that spinal diseases were the most frequent cause for hospital visits. U-code, a component of the KCD (the Korean version of International Classification of Disease), has been used as a specific diagnostic code system for KM doctors. Y.-S. Lee et al. redistributed U-codes into related KCD codes and estimated that the total burden of diseases in Korea, of which 2012 were KM treatments, were included. The authors found that when KM was included in the calculation for burden of disease, musculoskeletal disorders showed by far the most growth.

As with practice of alternative and complementary medicine in other countries, good results from clinical practice with KM for stubborn diseases are often reported, even in conditions where conventional medicines treatment strategies are not yet available. T. Park and S. Lee reported improved clinical outcomes (especially for recurrence) for urinary bladder cancer using complex KM interventions. J. Lee et al. using a retrospective observational approach assessed the effectiveness of the herbal decoction, Shihogyejitang, for 54 drug resistant epileptic children and found meaningful seizure reduction. While these studies do not provide conclusive evidence for the use of KM for these conditions, they do highlight the need for more rigorous clinical trials in the future.

We hope this special issue will be helpful for both researchers and TEAM practitioners who want to comprehend and know more about the current clinical status of KM in general practice.

*Tae-Hun Kim* *Tae-Hun Kim* *Christopher Zaslawski* *Christopher Zaslawski* *Sunoh Kwon* *Sunoh Kwon* *Jung Won Kang* *Jung Won Kang*

